# The Mediating Roles of Psychological Autonomy, Competence and Relatedness on Work-Life Balance and Well-Being

**DOI:** 10.3389/fpsyg.2019.01267

**Published:** 2019-05-29

**Authors:** Anestis Fotiadis, Khadija Abdulrahman, Anastasia Spyridou

**Affiliations:** ^1^College of Communication and Media Sciences, Zayed University, Abu Dhabi, United Arab Emirates; ^2^College of Business, Zayed University, Abu Dhabi, United Arab Emirates; ^3^Faculty of Management and Economics, Gdańsk University of Technology, Gdańsk, Poland

**Keywords:** well-being, work life balance, autonomy, competence, relatedness

## Abstract

The main objective of this current research is to investigate the impact of “work balance” on “psychological well-being” using employees within the hospitality industry in United Arab Emirates as statistical units. To meet the objective of this research, we developed a structural equation model to examine how psychological autonomy, psychological competence, and psychological relatedness affect psychological well-being and work-life balance, as well as the effect of work-life balance on psychological well-being. We also examine the mediating effect of work-life balance in these relationships. The results of this study show that psychological autonomy affect positively both psychological well-being and work-life balance, whereas psychological competence only affect psychological well-being positive. Moreover, psychological relatedness affects negatively both psychological well-being and work-life balance while work-life balance affects positively psychological well-being.

## Introduction

The hospitality industry is a labor-intensive environment that operates 365 days a year and around the clock and requires working hours that sometimes exceed the 40 h/week standard for full-time employment for the production of services. Such a working environment is highly problematic because employees must deal with a precarious and exploitative work environment, a low salary, anti-social working hours, and poor social status, all of which prevent them from balancing work, family duties, and other elements necessary for their well-being (Baum, 2019). Consequently, the working conditions within the hospitality industry make it impossible for employees to benefit from satisfactory levels of psychological needs, such as an increase in psychological well-being and a proper work-life balance (Haider et al., 2018). However, the quality of the employee-customer relationship is crucial (Castellanos-Verdugo et al., 2009), particularly in the hospitality industry, in which service employees are among the most relevant success factors (Tsai, 2009).

**FIGURE 1:**
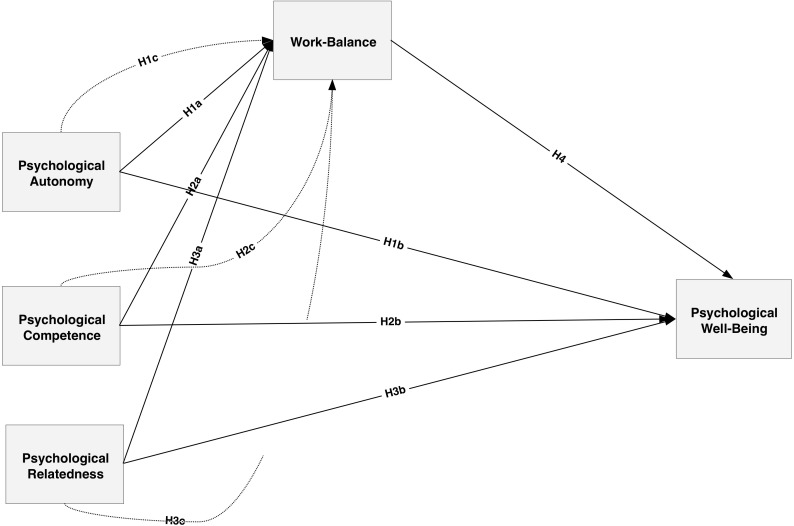
Proposed Research Model. Source: Self-Structure.

In contrast to a good work-life balance, work-life conflicts decrease employees’ well-being and increase psychological stress, which may lead to lower employee commitment and higher work-withdrawal behaviors (Hofmann and Stokburger-Sauer, 2017). Therefore, practitioners and researchers alike must understand the significance of a healthy and/or proper *work-life balance* and *psychological well-being* to improve working conditions, an important factor that helps to retain qualified employees. In addition to the importance of these psychological needs, empirical evidence regarding the link between *work-life balance* and *psychological well-being* as well as among *psychological autonomy*, *psychological competence*, and *psychological relatedness* to *well-being* is scarce in the marketing literature, particularly in the hospitality industry. To help fill this gap, the current research investigates the impact of psychological autonomy, psychological competence, and psychological relatedness on “work-life balance” and psychological well-being. This study also investigates the impact of work-life balance on “psychological well-being” and the mediating effect of work-life balance using employees within the hospitality industry in United Arab Emirates as statistical units.

## Literature Review

### Psychological Well-Being

According to Self-determination theory (SDT), vitality and effective functioning are two innate tendencies for which people generally aim once their basic psychological needs are met (Deci and Ryan, 2008). Basic needs are defined as “innate psychological nutriments that are essential for ongoing psychological growth, integrity, and well-being” (Deci and Ryan, 2000, p. 229). As such, non-optimal psychological outcomes are more likely to emerge (e.g., lower psychological growth and well-being) when basic needs are left unsatisfied (Deci and Ryan, 2000). SDT highlights autonomy, competence and relatedness as three psychological needs. Autonomy refers to the experience of volition and the approval of one’s behavior (DeCharms, 1968). However, competence defines the desires expressed by an individual to master their environment by enjoying valued outcomes within it (White, 1959), whereas relatedness defines the individual feeling of being connected to others (Baumeister and Leary, 1995).

According to Klainin-Yobas et al. (2016), psychological well-being (PWB) is an important factor that can improve health and increase life expectancy. Hedonism and eudaimonia are two types of PWB constructs constantly conveying the situation of the workplace environment (Joshanloo, 2018). Based on psychological well-being at the workplace, hedonism situates the extent to which employees can expand positive effects and decrease negative consequences, whereas eudaimonia situates the extent to which employees can become healthier and more joyful and prosperous in a particular work environment (Rahmani et al., 2018). As Horvath (2018) mentions, high-level construals have a higher impact on the long-term well-being of the individual. As posited by Self-Determination Theory, social environment plays an important role in satisfying basic psychological needs. Supportive social contexts may enhance the level of satisfaction of basic psychological needs with the consequential outcomes of optimal functioning and well-being, whereas a coercive social context hinders the satisfaction of these needs, leading to malfunctioning and ill-being (Ryan and Deci, 2000). Consistently, Trépanier et al. (2013) investigated how exposure to workplace bullying predicts psychological health at work. The authors found that mistreatment negatively predicts the level of work engagement, thereby leaving employees’ psychological needs for autonomy as well as for competence and relatedness unsatisfied.

### Work-Life Balance

The ability to balance both work and personal activities helps employees improve the quality of their well-being (De Cieri et al., 2002). Consequently, work-life balance initiatives must be designed strategically as well as accepted and respected within the organizational culture to adequately benefit employees and the organizations for which they work. To achieve such a health balance, employees must have the autonomy to fully enjoy life both inside and outside the workplace (Moore, 2007). Monetary benefits are often used as a motivational factor in the working settings. However, employees who value work-life balance are less likely to be motivated by the monetary benefits offered by their employers if these benefits do not add greater value to the quality of their lives.

Many empirical studies report an increase in employee’s productivity and motivation within organizational settings in which work-life balance initiatives are strategically implemented (Lazar et al., 2010). Similarly, the implementation of work-life balance programs has a strategic stance of addressing employee retention as a means of reducing costs and the burdens associated with recruitment and absenteeism. Similarly, initiating work-life balance programs has been supported as a means through which organizations improve labor recruitment, the level of performance and commitment of their employees, and their level of engagement (Hyman and Summers, 2007). A healthier work-life balance is associated with higher psychological well-being. Although the literature conveys the benefits of work-life balance for both employees and employers, quantifying such benefits remains challenging because of the qualitative nature of most of the work-life balance initiatives. Therefore, reliable measurements must be identified to help quantify the level of contribution, either directly or indirectly, of work-life balance to organizational outcomes to an evidence-based support in favor of such statements (Eby et al., 2005).

### Psychological Autonomy

“Autonomy refers to self-government and responsible control for one’s life” (Keller, 2015, p. 1). Inductively, autonomy determines the capacity of an individual to make informed and uncoerced decisions. In this respect, having autonomy also determines the extent to which an individual has control over their options and choices and meets their desires accordingly. Several authors (e.g., Benz and Frey, 2008) argued employee well-being is not only acquired through psychological autonomy but also through the employees’ capacity to accomplish unique goals within a particular workplace context as supported by self-determination helping them satisfy their psychological needs. Being free from taking non-coerced decisions at the workplace and feeling free to act in accordance with one’s own decisions are two different situations. From the perspective of self-determination theory in the workplace context, providing support for psychological autonomy at the workplace does not require relative autonomy to make uncoerced decisions. However, it grants employees the freedom of agency to make meaningful decisions and the freedom to engage willingly in activities that bring a high level of satisfaction to their psychological needs, rather than being the ultimate result of pressure or coercion from upper management (deCharms, 1981).

Several studies have discovered a positive association between psychological autonomy and other measures of psychological well-being, such as hedonic happiness (Sheldon et al., 1996), life satisfaction (Cordeiro et al., 2016), and subjective vitality. These findings lead us to posit people who enjoy higher levels of autonomy at the workplace are likely to experience greater well-being than those who experience constraint or lower autonomy. In such instances, the relationship between the enjoyment of greater well-being and higher levels of autonomy at the workplace will be mediated by psychological autonomy. Therefore, we hypothesize the following:

H1a: Psychological autonomy correlates positively to psychological well-being.
H1b: Psychological autonomy correlates positively to work-life balance.
H1c: Work balance has a mediating effect on the relationship between psychological autonomy and psychological well-being.

### Psychological Competence

Psychological autonomy effects positively psychological competence. Given such a positive effect, psychological competence is more likely to provide greater individual feelings of self-efficacy and personal mastery as well as enhancement of core competence within workplace settings offering a good fit of work-life balance. Most of the time, skilled employees are individuals seeking further development of their *psychological competence* at the workplace. Unfortunately, they often face uncertainty in fully satisfying this psychological need (McMullen and Shepherd, 2006). More importantly, highly skilled employees tend not to rely on routines but to seek ways to adapt their behaviors as they move through organizational ladders (McGrath and MacMillan, 2000). Ultimately, these employees tend to lean on workplace environmental settings to develop the core competencies necessary to satisfy their need for psychological competence and to ensure a good fit of work-life balance and psychological well-being in today’s highly competitive and constantly changing business environment. By facilitating and encouraging personal goal setting that does not conflict with work-life balance, managers may provide their employees the opportunities to further develop the skills and capabilities that can help them achieve both their personal objectives (e.g., satisfy their need for psychological well-being) and those of their organization (Haynie and Shepherd, 2011).

Several field and experimental studies have investigated the relationship between psychological competence, psychological well-being, and work-life balance. Consistently, psychological competence has been associated with greater psychological well-being and work-life balance. Similarly, work-life balance was found to mediate the relationship between psychological competence and psychological well-being. Valorizing psychological competence may bear significant benefits for employees and employers alike. For example, by satisfying the need for psychological competence, employees are more likely to increase their motivation for continuous capacity building. In turn, employers are likely to benefit from their employees’ acquisition of new knowledge and improvement of skills because their jobs are being done better and more effectually. Research further shows higher levels of psychology competence correlate positively to psychological well-being in addition to other psychological needs, such as hedonic happiness (Cordeiro et al., 2016), life satisfaction, and subjective vitality (Baard et al., 2004), etc. Based on the findings mentioned above, we hypothesize the following:

H2a: Psychological competence correlates positively to psychological well-being.
H2b: Psychological competence correlates positively to work-life balance.
*H2c: Work balance has a mediating effect on the relationship between psychological competence and psychological well-being*.

### Psychological Relatedness

Psychological relatedness refers to the social nature of human beings and their connectedness with others. Researchers agree psychological relatedness is significant within a supportive workplace setting in which closeness with others and social actions are valued. Being new at the workplace may produce the feeling of social isolation and loneliness (Gumpert and Boyd, 1984). Similarly, an unsupportive work environment can create a relationship gap between and within a hierarchy, resulting in feelings of remoteness, loneliness and social isolation (Hannafey, 2003). Because of the requirements for employees to commit to customers and co-workers, the feeling of remoteness may also be created even in a supportive workplace environment (Wood and Rowe, 2011; Shir et al., 2018). Consequently, a deficit of belonging may occur because of the negative emotions (e.g., grief, fear of failure, stress, and loneliness) often associated with the feeling of being isolated (Shepherd and Haynie, 2009).

In contrast to the sentiment of feeling alienated or marginalized, autonomy or supportive autonomy at the workplace is conducive to giving employees the feeling of being connected with their co-workers (Baumeister and Leary, 1995). Consequently, employees may enjoy the freedom to organize their own approach to achieving organizational objectives and the freedom to interact with individuals and connect to professional networks relevant to their personalities. When employees have the autonomy to establish relationships and interact freely with individuals of their choice, employees are more likely to devote time to nurture the relationships and professional networks in which they have been encouraged to engage. Managers may protect those working under their supervision from developing feelings of being socially isolated (Francis and Sandberg, 2000). Otherwise, employees may experience feelings of social isolation when they are obliged to abide by the decisions of others, are pressured to collaborate with individuals chosen by upper management, and are forced to follow organizational rules and routines (Simon, 1991).

Since a supportive autonomy at the workplace confers better opportunities for individuals to consolidate relationships at work, they are more likely to experience greater feelings of connectivity with their peers, which may result in a satisfactory level of well-being. Positive feelings of psychological relatedness are strongly correlated with other psychological needs, such as psychological relatedness as well as subcategories of psychological needs, such as well-being, hedonic happiness, subjective vitality, and life satisfaction (Sheldon et al., 1996; Baard et al., 2004; Cordeiro et al., 2016). Consequently, the following are hypothesized:

H3a: Psychological relatedness correlates positively to psychological well-being.
H3b: Psychological relatedness correlates positively to work-life balance.
H3c: Work-life balance correlates positively to psychological well-being.
H4: Work-life balance mediates the relationship between psychological relatedness and psychological well-being.

## Methodology

### Measurement

To meet the objective of this study, we developed a quantitative questionnaire survey, and a written informed consent was obtained from participants before they completed the questionnaire. The questionnaire was divided into six sections. The first section collected basic socio-demographic information. The five remaining sections were designed to assess the relationships between each of the independent variables of this study: psychological autonomy, psychological competence, psychological relatedness, the mediating variable “work-life balance,” the dependent variable “psychological well-being,” and the mediating effect of “work-life balance.” Then, a Full Ethical Clearance was obtained from Zayed University Research Ethics Committee on the 6th of March 2019 (ZU19_019_F). Items were measured using a five-point Likert scale.

### Data Collection

Data were collected from two sources: online and onsite. From the online source, 361 responses were collected. For the onsite source, 220 valid responses were collected using the traditional paper-pencil survey technique at hospitality sites in Dubai, United Arab Emirates. A summary of the basic respondent information demonstrates that, among the 581 respondents who completed the questionnaire survey, 59.7% were female and 41.3%, male. The results also show 42% of respondents were in the age bracket of 19–29 years old. Furthermore, 22.4% of the respondents reported they were university students, while 52.4% were employed full time. The academic credentials of the respondents accounted 41.6% for bachelor’s degrees and 24.1% for high school degrees.

### Data Analysis

This study intends to understand the impact of “work-life balance” on “psychological well-being” using employees within the hospitality industry in United Arab Emirates. To meet this objective, a structural equation model was developed to survey how psychological autonomy, psychological competence, and psychological relatedness affect psychological well-being and work-life balance. The mediating effect of work-life balance in these relationships is studied. Items were analyzed for convergent validity, reliability, and discriminant validity. The reliability of the items was confirmed with a Cronbach’s alpha test and validity with a confirmatory factor analysis (CFA) using SPSS version 25.0. Model fitness was tested using structural equation modeling (SEM). 4.1. Research design and sample.

## Results

### Validity and Reliability Analysis and Model Fit

We tested the scale of the items on the questionnaire for convergent validity, reliability, and discriminant validity. The assessment of convergent validity of latent variables generally requires a loading threshold equal to or above 0.60. The convergent validity of the measurement of our model was confirmed, with each item yielding a value higher than the minimum threshold generally required. To assess the reliability of the scale of the items, we used composite reliability (CR), Cronbach’s alpha, and average variance extracted (AVE). The CR and Cronbach’s alpha values for each item were above the threshold value of 0.7, and AVE was above the threshold of 0.5, as suggested by Bagozzi and Yi (1988). Since these measurements meet the recommended threshold values, we concluded our model was reliable. We also conducted a discriminant validity test using a correlation matrix, as shown in Table 1, to ensure the square root of the AVE for each construct was greater than the inter-construct correlations.

**Table 1:** Loading, AVE and composite reliability (CR) values.

Item	Mean	SD	Loading	SE	AVE	CR
Psychological autonomy	5.63	1.72			0.86	0.87
PA1			0.87	0.76		
PA2			0.66	0.61		
PA3			0.65	0.60		
PA4			0.86	0.74		
PA5			0.98	0.96		
PA6			0.95	0.90		
PA7						
Psychological competence	5.02	1.77			0.83	0.85
PC1			0.95	0.90		
PC2			0.91	0.83		
PC3			0.89	0.79		
PC4			0.87	0.76		
PC5			0.84	0.77		
Psychological relatedness	5.34	1.69			0.87	0.91
PR1			0.83	0.69		
PR2			0.91	0.83		
PR3			0.86	0.74		
PR4			0.87	0.76		
PR5			0.84	0.70		
PR6			0.94	0.82		
Work balance	5.31	1.72			0.79	0.83
WB1			0.91	0.83		
WB2			0.90	0.82		
WB3			0.87	0.72		
Psychological well-being	4.72	1.95			0.70	0.88
PWB1			0.72	0.67		
PWB2			0.83	0.74		
PWB3			0.85	0.70		
PWB4			0.82	0.76		
PWB5			0.76	0.74		
PWB6			0.81	0.67		


The results for the goodness of fit index (GFI) = 0.93, normed fit index (NFI) = 0.92, and comparative fit index (CFI) = 0.95, showing an overall good fit for our model when compared to the suggested threshold of 0.90. The results also yield an adjusted goodness of fit index (AGFI) of 0.89, which is higher than the suggested 0.80. Similarly, the root mean square error of approximation (RMSEA) yields a good fit of 0.08. Furthermore, we tested the hypotheses of this study using SEM AMOS. The results for Standardized Beta Coefficients and the *T*-Value are presented in Table 2. The results indicate psychological autonomy has a positive effect on psychological well-being (β = 0.12), and on work-life balance (β = 0.19). Psychological competence has a positive effect on psychological well-being (β = 0.23), but has a negative effect on work-life balance (β = -0.14). However, the results show psychological relatedness has a negative effect on both psychological well-being (β = -014) and work-life balance (β = -0.23). For hypothesis H_4_, work-life balance has a positive effect on psychological well-being, with a Standardized Beta Coefficient of 0.09. As shown in Table 2, four (4) of seven (7) hypotheses described in our model are confirmed through statistically significant path coefficients.

**Table 2:** Standardized beta coefficients and hypotheses support.

H1–H9	Variables in the paths model	*β*^∗^	*T-Value^∗∗^*	
H_1a_	Psychological Autonomy → Psychological Well-Being	0.12	2.1	Supported
H_1b_	Psychological Autonomy → Work-life Balance	0.19	2.13	Supported
H_2a_	Psychological Competence → Psychological Well-Being	0.23	2.03	Supported
H_2b_	Psychological Competence → Work-life Balance	-0.14	-1.76	Not Supported
H_3a_	Psychological Relatedness → Psychological Well-Being	-0.14	-0.81	Not Supported
H_3b_	Psychological Relatedness → Work-life Balance	-0.23	-3.21	Not Supported
H_4_	Work-life Balance → Psychological Well-Being	0.09	0.98	Supported


The model indicates a high level of predictive power of the independent variables, which accounts for 37% (R^2^) of the variance in the dependent variable (psychological well-being).

### Mediating Effects

In the next step, we assess the mediating effect of work-life balance in the relationship between each independent variable and the dependent variable. Psychological autonomy significantly and positively affects work balance with a regression weight equal to 0.718 and a *P*-value less than 0.001. Work-life balance significantly affects psychological well-being with a regression weight of 1.07. However, the results indicate a decrease of the causal effect of psychological autonomy on psychological well-being. Such a decrease in the regression weight indicates a non-mediating effect of work-life balance (Hayes et al., 2011). The mediating effect of work-life balance on the relationship between psychological competence and psychological well-being was tested. Different from what was hypothesized, the results show that work-life balance is not a valid mediating factor in this relationship. Similarly, we tested the mediating effect of work-life balance in the relationship between psychological competence and psychological well-being, then between psychological relatedness and psychological well-being. The results confirm the mediating of work-life balance in the relationship both between psychological competence and psychological well-being, and between psychological relatedness and psychological well-being.

## Discussion

The relevance of psychological autonomy was found to be positively associated with psychological well-being and work-life balance. Similarly, psychological competence is positively associated with psychological well-being. These results are all supported by the literature (Sheldon et al., 1996; Baard et al., 2004; Moore, 2007; Lazar et al., 2010; Cordeiro et al., 2016). Differently from what was hypothesized based on other findings in the literature, psychological competence is negatively associated with work-life balance. Moreover, we found that psychological relatedness is negatively associated with both psychological well-being and work-life balance, whereas work-life balance is positively associated with psychological well-being. Thus, a proper interpretation of these results can be performed only by considering the social and legal contexts, the demography of the workforce, and the nature of the industry in which these variables were studied.

The hospitality industry is a highly workforce-intensive sector requiring the recruitment of both unskilled and skilled labor for service provision. As such, managers are challenged to make the hospitality business more attractive to employees by tapping into their different psychological needs as a means of reducing turnover to meet the demand of the boom in the tourism industry in a country. Given today’s workforce trends, however, employees are more inclined to prioritize successful career objectives, which conflicts with other aspects of their lives. In addition to this trend, psychological needs may vary among individuals with regards to age, level of education, and citizenship status (United Arab Emirates in our case). Individuals with adequate credentials tend to expect more autonomy over their job. Regarding the trends of prioritizing the pursuit of career objectives, younger people with adequate levels of education emphasize more their psychological autonomy and competence as a means of achieving both psychological well-being and work-life balance, which partially situates their paradigm of pursuing successful career objectives. Therefore, these individuals may feel satisfied with sustainable human development initiatives supporting their ultimate career objectives. Psychological autonomy and psychological competence are highly related. However, the reasons remain unclear why psychological autonomy positively influences work-life balance, whereas psychological competence moves in the opposite direction. However, the negative association between psychological competence and work-life balance seems logical for employees who voluntarily assign importance to their career objectives over other aspects of their lives.

Satisfying psychological needs is vital for a healthy and productive workplace environment. However, the initiative of supporting or designing a strategic plan to meet these needs may not yield the expected results, as indicated in this study. Consequently, researchers and managers alike need to understand that each psychological need varies among individuals with regards to their purpose in life, their financial situation, their familial situation, and social and legal contexts. Additionally, age and education should be considered when addressing the significance of these psychological needs. Therefore, future research may consider investigating how the differences in age, social status, and legal context influence psychological needs initiatives in workplace settings. Furthermore, the ways individuals interpret their psychological needs and their expectations regarding these needs are worthy of deep investigation. The study was limited to the hospitality industry in United Arab Emirates. Therefore, a similar result is not likely to be found within other industries, given the particular characteristics of the hospitality industry. Additionally, differences in legal contexts and the characteristics of the labor workforce vary by country. This variance may also explain the difficulty in making plausible comparisons regarding the states of psychological needs and their implications for employees and businesses in different cultural and legal settings.

## Data Availability

The datasets for this manuscript are not publicly available because they contain info that is not allowed to be publicly available based to the agreement we sign. Requests to access the datasets should be directed to Anestis.Fotiadis@zu.ac.ae.

## Ethics Statement

Zayed University Ethics Committee. Full Ethical clearance was given for this study: ZU19_019_F.

## Author Contributions

All authors listed have made a substantial, direct and intellectual contribution to the work, and approved it for publication.

## Conflict of Interest Statement

The authors declare that the research was conducted in the absence of any commercial or financial relationships that could be construed as a potential conflict of interest.
